# Frame Coating of Single-Walled Carbon Nanotubes in Collagen on PET Fibers for Artificial Joint Ligaments

**DOI:** 10.3390/ijms21176163

**Published:** 2020-08-26

**Authors:** Alexander Yu. Gerasimenko, Natalia N. Zhurbina, Nadezhda G. Cherepanova, Anna E. Semak, Vadim V. Zar, Yulia O. Fedorova, Elena M. Eganova, Alexander A. Pavlov, Dmitry V. Telyshev, Sergey V. Selishchev, Olga E. Glukhova

**Affiliations:** 1 Institute of Biomedical Systems, National Research University of Electronic Technology MIET, Shokin Square 1, Zelenograd, 124498 Moscow, Russia; natalia93zhurbina@gmail.com (N.N.Z.); fedorovauo@mail.ru (Y.O.F.); telyshev@zitc-mt.ru (D.V.T.); selishchev@bms.zone (S.V.S.); 2 Institute for Bionic Technologies and Engineering, I.M. Sechenov First Moscow State Medical University, Bolshaya Pirogovskaya street 2-4, 119991 Moscow, Russia; 3 Department of Morphology and Veterinary Expertise, Russian State Agrarian University—Moscow Timiryazev Agricultural Academy, Timiryazevskaya street 49, 127550 Moscow, Russia; ncherepanova75@mail.ru (N.G.C.); semakq@gmail.com (A.E.S.); 4 Department of Traumatology and Orthopedics, M.F. Vladimirskii Moscow Regional Research and Clinical Institute, Shepkina street 61/2, 129110 Moscow, Russia; vzar@list.ru; 5 Research Laboratory of Promising Processes, Scientific-Manufacturing Complex “Technological Centre”, 1-7 Shokin Square, 124498 Moscow, Russia; 6 Micro- and Nanosystems Research and Development Department, Institute of Nanotechnology of Microelectronics of the Russian Academy of Sciences, 32A Leninsky Prospekt, 119991 Moscow, Russia; eganovaem@mail.ru (E.M.E.); pavlov.a@inme-ras.ru (A.A.P.); 7 Department of Physics, Saratov State University, Astrakhanskaya street 83, 410012 Saratov, Russia

**Keywords:** nanostructured materials, coating, single-walled carbon nanotubes, collagen, IR radiation, PET fibers, artificial knee ligaments, bone regeneration, biodegradation, osteoconductivity, hemocompatibility

## Abstract

The coating formation technique for artificial knee ligaments was proposed, which provided tight fixation of ligaments of polyethylene terephthalate (PET) fibers as a result of the healing of the bone channel in the short-term period after implantation. The coating is a frame structure of single-walled carbon nanotubes (SWCNT) in a collagen matrix, which is formed by layer-by-layer solidification of an aqueous dispersion of SWCNT with collagen during spin coating and controlled irradiation with IR radiation. Quantum mechanical method SCC DFTB, with a self-consistent charge, was used. It is based on the density functional theory and the tight-binding approximation. The method established the optimal temperature and time for the formation of the equilibrium configurations of the SWCNT/collagen type II complexes to ensure maximum binding energies between the nanotube and the collagen. The highest binding energies were observed in complexes with SWCNT nanometer diameter in comparison with subnanometer SWCNT. The coating had a porous structure—pore size was 0.5—6 μm. The process of reducing the mass and volume of the coating with the initial biodegradation of collagen after contact with blood plasma was demonstrated. This is proved by exceeding the intensity of the SWCNT peaks G and D after contact with the blood serum in the Raman spectrum and by decreasing the intensity of the main collagen bands in the SWCNT/collagen complex frame coating. The number of pores and their size increased to 20 μm. The modification of the PET tape with the SWCNT/collagen coating allowed to increase its hydrophilicity by 1.7 times compared to the original PET fibers and by 1.3 times compared to the collagen coating. A reduced hemolysis level of the PET tape coated with SWCNT/collagen was achieved. The SWCNT/collagen coating provided 2.2 times less hemolysis than an uncoated PET implant. MicroCT showed the effective formation of new bone and dense connective tissue around the implant. A decrease in channel diameter from 2.5 to 1.7 mm was detected at three and, especially, six months after implantation of a PET tape with SWCNT/collagen coating. MicroCT allowed us to identify areas for histological sections, which demonstrated the favorable interaction of the PET tape with the surrounding tissues. In the case of using the PET tape coated with SWCNT/collagen, more active growth of connective tissue with mature collagen fibers in the area of implantation was observed than in the case of only collagen coating. The stimulating effect of SWCNT/collagen on the formation of bone trabeculae around and inside the PET tape was evident in three and six months after implantation. Thus, a PET tape with SWCNT/collagen coating has osteoconductivity as well as a high level of hydrophilicity and hemocompatibility.

## 1. Introduction

The total number of arthroscopic operations for replacing the knee joint ligament increases every year, along with a rise in the number of repeated operations [1,2]. The medial and cruciate ligaments are stabilizers of the knee joint, especially the anterior cruciate ligament. Damage to the anterior cruciate ligament (ACL) is the most common and accounts for up to 50% of the total number of joint injuries [3]. The majority of patients are young people who lead an active lifestyle and play sports. In case of damage to the ligaments, a violation of the joint functionality and limitation of limb mobility occur [4]. Against this background, concomitant diseases such as joint instability, synovitis, gonarthrosis develop, which can lead to disability [5].

In most cases, the injured ligaments are not capable of self-repair. The reason for this is considered imperfect cell homeostasis—immature fibroblasts that cannot form a connective tissue scar at the site of rupture without the preliminary formation of the fibrin framework are produced in the area of ligament rupture [6,7]. Synovial fluid of the knee joint prevents the formation of such a framework [8]; therefore, the healing of ligament rupture and restoration of joint functionality does not occur. The only effective method for treating damage to the ligaments of the joints is currently considered to be arthroscopic surgery with a complete replacement.

The native connection of ligaments and tendons with bone is a highly specialized and organized tissue that transfers mechanical stress from soft tissue to bone. The junction region comprises four types of tissues—ligament, fibrous cartilage, mineralized fibrous cartilage, and bone [9,10]. The structure and composition of the native compound of the ligament and bone are not completely restored after damage, which leads to mechanical and structural disturbances [10]. The healing of the ligament-bone joint area is slower than that of the bone-bone joint area due to weak vascularization of the fibrous cartilage and loss of bone tissue in the lesion area [11,12].

Ligament plastic surgery is performed using the patient’s own tissues (autograft), donor tissues (allograft), or synthetic implants. The choice of material depends on the general condition of the patient and the severity of the injury. The number of tissues for autografts in the body is extremely limited. More than 34% of cases of using auto- or allografts are associated with poor implant engraftment and the need for repeated surgery [13,14]. When reconstructing ACL using autografts, pain in the knee, a decrease in the range of motion, and muscle weakness often occur [15,16]. Also, the use of an allograft is associated with an increased risk of developing the body’s immune response to a foreign body and transmission of infections from the donor to the recipient [16].

Synthetic implants are devoid of the main disadvantages of auto- and allografts—they do not cause an immune response, avoid damage to healthy tissues during the collection of donor material, and do not require long preoperative preparation. In addition, they have high mechanical characteristics.

Currently, the most common synthetic ligament implant is LARS (Ligament Augmentation Reconstruction System; Surgical Implants and Devices, France). LARS is composed of polyethylene terephthalate (PET) fibers [17]. PET implants contribute less to the development of synovitis than polyester, polytetrafluoroethylene, or polypropylene [18].

However, the results of clinical trials of synthetic PET implants do not provide unambiguous information on their effectiveness in the early period after surgery [19]. The problem is the cellular response in the intraarticular part to PET [20]. Polyethylene terephthalate is a hydrophobic and chemically stable, bioinert material, with low biological properties [20]. In the case of the native ligament between the soft tissues of the ligament and the bone, there is a transition layer of the fibrocartilage transitional zone. However, when using PET ligaments between the implant and the bone channel wall, a fibrous scar is observed [11,21]. The scar has very low mechanical parameters and has a strong negative effect on implant stability in the bone channel [22]. This is the reason for the development of cone deformation in the bone channel during the interaction with a synthetic ligament implant. As a result, a big problem arises due to the instability of the joint and the unsatisfactory result of the operation in the short term, which will greatly affect the outcome of the operation in the long term.

Reliable fixation of the ligament implant in the bone channel in the early period after surgery is very important [23,24]. The success of the bone channel healing is a fundamental factor affecting the result of the reconstruction of the ACL. The quality of tissue repair at the implant/bone border determines how fast and complete the early and late restoration of joint stability will be.

In the past decade, strategies to accelerate and increase the efficiency of osseointegration of the ligament implant in the bone channel have been developed, including alternative methods of implant fixation [1], and various biological coatings that increase the functionality and biocompatibility of the implant [25]. The use of biocompatible or bioactive coatings is aimed at increasing the hydrophilicity of the implant and, thereby, enhancing the formation of bone tissue and reducing the size of the fibrous scar at the ligament-bone interface. To change the properties of the implant surface, the coating should contain biocompatible, cytophilic, and osteoinductive molecules or natural hydrophilic polymers [26,27], as well as suitable surface topography. These factors positively affect the proliferation and differentiation of cells and, accordingly, the formation of the extracellular matrix and its mineralization [28].

Coatings based on bioceramics, in particular, hydroxyapatite, are widely used [29,30]. Due to their biocompatibility and osteoconductive properties, hydroxyapatite-based coatings have been used for a long time to enhance bone growth at the border between the implant and the bone channel wall [30,31]. The use of hydroxyapatite is limited by its predisposition to the formation of agglomerates when applied to the PET and, as a result, the deteriorated properties of the coating [31].

Bioactive glass is another biomedical material with high osteoconductivity and bioactivity [32]. Bioactive glass is involved in the healing of bone tissue by the release of calcium and silicon ions, which stimulate the proliferation and differentiation of osteoblasts [33]. However, a simple soaking of the implant in a solution of bioactive glass does not ensure the formation of a uniform and stable coating and reliable binding of the coating to PET fibers, which limits the use of this method [34].

When a composite coating of hydroxyapatite and bioactive glass is applied to a synthetic ligament implant, the osteoconductive properties of the implant are enhanced [35,36]. The combination of these two components significantly improves cell survival and stimulates the cellular activity of alkaline phosphatase in comparison with pure hydroxyapatite [37]. Also, it improves protein adsorption, cell attachment, and proliferation [38]. The application of this method is also limited by the complexity of forming a uniform and stable coating with high adhesion to the implant.

Polymers are widely used to modify various implants, including joint ligaments. Their chemical composition and structure are largely reminiscent of living tissue, in addition, they have excellent biological properties. As organic coatings containing various functional organic biomolecules, hydroxypropyl cellulose having hydrophilic and bioadhesive properties is used [20]; hyaluronic acid, providing biocompatibility and hydrophilicity of the implant surface [39]. Studies of these coatings showed some improvement in osseointegration and bone healing, but bone formation was not complete. In addition, in accordance with histological images, there was no direct contact of the newly formed bone with the original native bone [40].

The positive effect of a coating based on cationized gelatin and hyaluronic acid on the inhibition of inflammatory cell infiltration and osseointegration of a ligament implant in the distal area of the bone channel has been shown [41,42]. The disadvantage of using such a coating is the slow process of osseointegration, still associated with the formation of a thin layer of scar tissue at the implant-bone border.

Despite the fact that the structure and biomolecular composition of polymers are close to the structure and composition of living tissues, they have low mechanical properties. A possible solution to this problem is to use a combination of polymers with a reinforcing component, for example, ceramics, metal, or carbon nanoparticles [43,44,45].

Carbon nanotubes (CNTs) have great potential in the field of tissue engineering, in particular, for enhancing biodegradable polymer frames, which are highly demanding in terms of the biomechanical characteristics of the designed structures [46,47,48,49].

Coatings based on chemically cross-linked CNTs of various types showed a positive effect on the osteogenic differentiation of human stem cells [47]. The additional functionalization of CNTs by carboxyl, carbonyl, or amine functional groups, synthetic (polyethylene glycol) or natural (collagen) polymers can affect the physicochemical properties of the material [50,51], its bioinertness, and improve biocompatibility [52]. It was proved by molecular methods simulations [53] and was confirmed in experiments in vitro [54,55] and in vivo [55].

The addition of CNTs to a matrix of a copolymer of lactic and glycolic acids allows obtaining a composite material for bone tissue engineering. Such material showed an improvement in the mechanical parameters of the polymer, as well as an increase in the adhesion of osteoblasts, their growth, and osteogenic differentiation [45].

To strengthen polylactide, graphene nanoplates and carbon nanotubes were used [46]. These materials were chosen because of the high mechanical strength that arises due to the strong bonds between carbon atoms. It was proved that the mechanical properties of polymers are improved due to the inclusion of carbon nanoparticles, as well as the absence of acute cytotoxic effects of the obtained nanocomposite material in vitro [56]. Coatings for bone implants based on carbon nanotubes and graphene materials enhance osteogenesis and regulate bone matrix mineralization in populations of human mesenchymal stem cells [57,58].

Nevertheless, at the moment, there are no comprehensive physico-biological and medical data (in vivo) on the behavior of PET artificial joints with composite coatings of biopolymers with carbon nanotubes in bone tissue. Such studies are important in the long-term. This is necessary for a better understanding of the tissue repair processes of the bone channel in conjunction with the degradation processes of the coating to fix the artificial ligament in the bone. In this regard, the authors developed a new method of deposition and coating composition based on ultra-pure single-walled carbon nanotubes (SWCNT) in a type II collagen matrix to modify the surface of an artificial bundle of woven PET fibers. The aim of the study was to investigate the effect of such a coating on the healing quality of the bone channel of laboratory animals (rabbits) during the implantation of an artificial ligament. It is assumed that the SWCNT/collagen coating will contribute to the formation of dense connective tissue (collagen fibers) [51,54,59] and new bone tissue [60,61] at the implant-bone interface compared with the control group without SWCNT for six months of observation.

## 2. Results

### 2.1. Modeling the Interaction of the SWCNT/Collagen Complex

**Figure 1:**
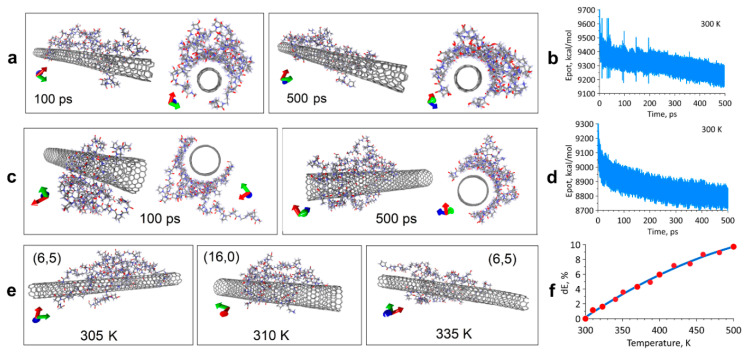
Molecular-dynamic empirical study of the formation process of the equilibrium configuration of the SWCNT/collagen complex: (**a**) Complex with SWCNTs (6,5) at 300 K; (**b**) Potential energy graph for the SWCNTs (6,5); (**c**) Complex with SWCNTs (16,0) at 300 K; (**d**) Potential energy graph for the SWCNTs (16,0); (**e**) Structures of complexes at various temperatures exceeding 300 K; (**f**) The change in the relative value of potential energy with increasing temperature.

A theoretical study on the mechanisms of forming the composite coating, namely, binding of SWCNT with collagen in an aqueous medium by the method of molecular dynamics, was made. The molecular dynamics simulation stage using the Assisted Model Building with Energy Refinement (AMBER) force field should ensure that the equilibrium configuration of the SWCNT/collagen complex is detected at different temperatures and for different types of SWCNT. A series of 20 numerical experiments was carried out on the formation of the SWCNT/collagen type II complex at temperatures in the range 300–500 K. Some simulation results at 300 ± 3 K are also presented (Figure 1). Figure 1a shows the complex models based on SWCNT (6,5) obtained during the first 100 and 500 ps. Figure 1b shows the dependence of the potential energy of this complex in the first 500 ps of the simulation. If a certain “loosening” of collagen in the first 100 ps was observed, then in a few hundred ps, the atomic structure of the SWCNT/collagen complex began to tend to its equilibrium state. This is clearly seen in Figure 1a,b. Collagen filaments covered the entire length of the SWCNT and wrapped it around the circumference. At the same time, the potential energy of the Epot decreased markedly and gradually tended to its equilibrium value. It should be noted here that a lower value of potential energy corresponds to the most stable and energetically favorable state of the complex. A similar situation is observed for the complex model based on SWCNT (16,0). In the first 100 ps, the collagen filaments formed a clot on the SWCNT (Figure 1c), but in the further 400 ps, the collagen took the form of an ordered structure tightly wrapping around the nanotube. This is also evidenced by a stable decrease in potential energy (Figure 1d). Indeed, the first ~ 0.5 ns are decisive, and it is precisely at this time that a significant restructuring of the complex occurs. In a period of time up to 1 or more nanoseconds, only a slight change in the atomic structure could be observed without any rearrangements. The equilibrium configurations of the complexes at some other temperature values are presented in Figure 1e, where complexes with SWCNT (6,5) at 305 K and 335 K are visible, as well as a complex with SWCNT (16,0) at 310 K. Even visually, it could be noted that already at 335 K, the spirals of collagen filaments begin to unfold, which means the onset of the denaturation process. This is also evidenced by the increase in the potential energy of the complexes.

Figure 1f shows the dependence of the relative change in potential energy dE with increasing temperature. The value of dE was calculated by the formula (Epot—Epot0)/Epot0, where Epot0 is the energy at a temperature of 300 K, and Epot is the potential energy at a given temperature. Based on these studies, it can be preliminary concluded that an increase in temperature inevitably leads to an increase in the potential energy of the equilibrium structure. This is easily explained by the general “loosening” of the atomic structure of collagen with increasing temperature. However, this behavior of potential energy does not provide reliable information about the interaction energy (bond) of a nanotube with collagen—Einter. The nature of the change in the Einter with increasing temperature is unknown, and to determine it, it is necessary to use more accurate methods—quantum, as mentioned above.

**Figure 2:**
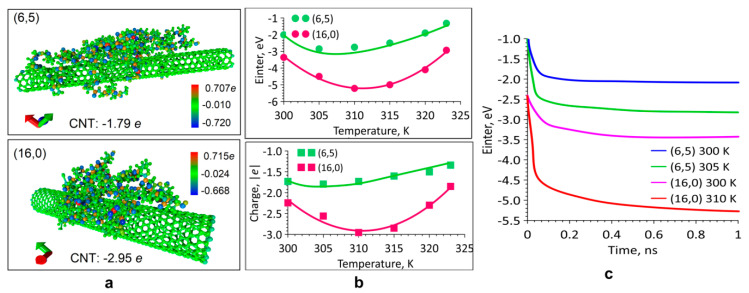
The binding energy of the SWCNT/collagen II complex and charge transfer: (**a**) Distribution of electron charge density; (**b**) Graphs of bonding energy and charge at SWCNT; (**c**) Graphs of the change in binding energy over time.

The next step was the study of the self-consistent charge density functional tight-binding (SCC DFTB) quantum method: atomic structure of complexes; electronic structure, and transfer of electronic charge; SWCNT—collagen bonding energies; the nature of the change in the binding energy with time. The complexes obtained at the previous stage at 300–323 K were investigated since it is precisely at these values that collagen denaturation does not occur. The atomic structure of the complexes was optimized at 300, 305, 310, 315, 320, and 323 K. A charge transfer was detected from collagen to a nanotube. Figure 2a shows two complexes with electron density distribution. Atomistic models correspond to the equilibrium configurations (Figure 1e) obtained at a temperature of 305 K and 310 K. The entire surface of the tube has a negative charge. More charges transferred to the tube (16,0) than to the tube (6,5). This determines the greater value of the bonding energy for the complex with the tube (16,0). The Einter bonding energies are shown in Figure 2b. According to the results of quantum modeling, the optimal temperature values for both types of complexes were revealed. It was found that in the case of thin SWCNT (6,5), the optimal temperature was 305 ± 0.5 K when the bonding energy was 2.8 ± 0.01 eV. In the case of tube (16,0), the temperature was higher than 310 ± 1 K, and the bonding energy was larger in the absolute value and was 5.4 ± 0.02 eV. With a further increase in temperature, the bonding energy gradually decreased. Based on the analysis of the data obtained, it could be concluded that for complexes based on SWCNT (6,5), the most optimal temperature range was 302–315 K for complexes based on SWCNT (16,0) the temperature range was another 305–317 K. At the indicated values, the temperature observed the highest values of bonding energy. Note that the largest charge transfer also corresponds to these temperature values (Figure 2b). For optimal temperature values and for a value of 300 K, the nature of the change in the bonding energy during the formation of equilibrium complexes was revealed. The graphs are shown in Figure 2c. All cases are characterized by rapid changes in the bonding energy in the first 100–200 ps, and then the bonding energy slowly changes, tending to a certain value corresponding to the equilibrium structure.

### 2.2. Structure of the Coating

**Figure 3:**
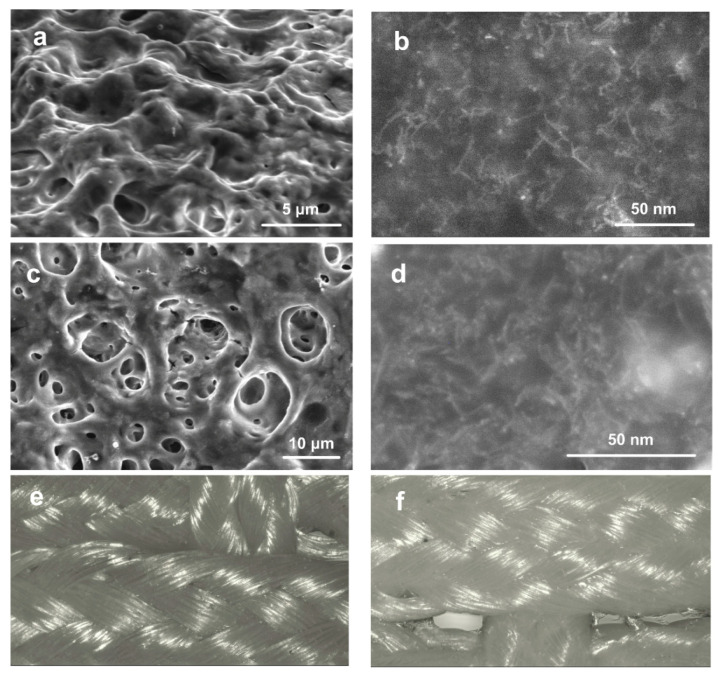
SEM images of (**a**) SWCNT/collagen coating surfaces and (**b**) an enlarged image of the SWCNT frame structure in the collagen matrix; (**c**) The surfaces of the SWCNT/collagen coating; (**d**) SWCNT frame structure in the collagen matrix after long-term contact with blood plasma; (**e**) Optical microscopy of the coating SWCNT/collagen before and (**f**) after long-term contact with blood plasma.

The SWCNT/collagen coating on the PET tape fibers had a hilly surface with a large number of pores (Figure 3a). We considered the results of three repeated measurements for five samples. The pore sizes ranged from 0.5 to 6 μm. The coating thickness was 20 ± 1 μm. On an SEM image with a large zoom, the isolated SWCNTs and their bundles are visible. The bundle formation is inevitable as Van der Waals forces bond the majority of SWCNTs along their entire length. Figure 3b shows the remote location of the SWCNTs from each other, compared to the original SWCNTs located close to each other (lying on top of each other) (Figure 3a). This is because the collagen biopolymer acted as a surfactant for SWCNT in dispersion.

### 2.3. Weight Loss

After thermal cycling, structural changes were compared with the initial state of the samples. Optical microscopy images showed practically no changes in the appearance of the coating on the fibers, except for a change in the sample color from dark to lighter (Figure 3e,f). This may indicate a decrease in the coating thickness after its interaction with blood serum during cooling and heating. The SEM image with the coating structure after thermal cycling shows a significant increase in the number of pores and their sizes up to 20 μm. At the same time, the internal structure of the SWCNT frame slightly changed. The nanotube frame became more visible due to a decrease in the volume of the collagen matrix (Figure 3d). No structural changes in the coatings in the form of faults, cracks, and sections of PET fibers without a frame coating were found. Studies of changes in coating mass were recorded for 10 samples. The relative weight loss of the coating after thermal cycling was 55–62%.

**Figure 4:**
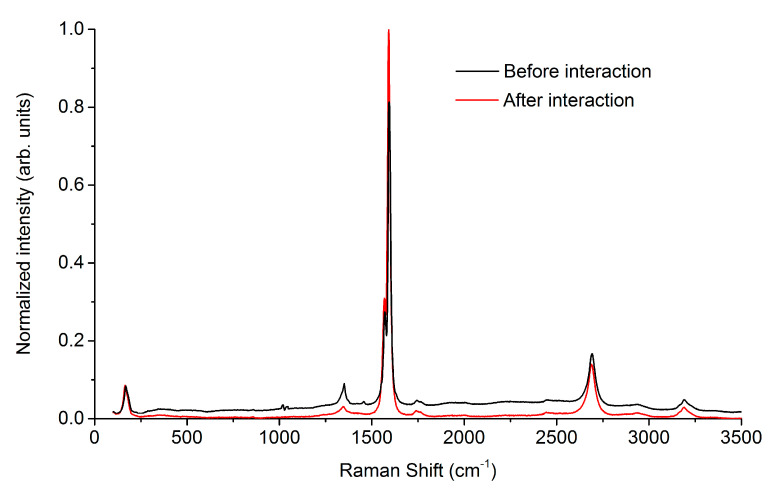
Raman spectra of the initial coating of SWCNT/collagen (black line) and (red line) after the implant was in the blood plasma during thermal cycling.

The Raman spectrum allows comparing the structural features of the coating before (black line) and after long-term contact with the plasma (red line). Figure 4 shows the Raman spectrum, averaged over the five samples each scanned in three points, and then normalized by the G mode (G = 1).

Firstly, we can say that the formation of collagen-based coatings by a spin coater and IR source basically does not violate its structure. This is evidenced by standard vibrational collagen bands. Peaks of about 1010 cm^−1^ and 1037 cm^−1^ relate to phenylalanine (aromatic alpha-amino acid). A peak at about 1455 cm^−1^ relates to methyl and methylene—deformation vibrations δ (CH_3_ and CH_2_). A peak at about 1263 cm^−1^ corresponds to the vibration of Amide III, which is used to assess the the conformational characteristics of the protein backbone. The low intensity of the peak in the spectrum means that the structural integrity was modified. Presumably, conformational changes are associated with the formation of a bulk matrix of collagen, which acts as a link in the formation of the carbon nanotubes frame. The peak in the range of 1640–1650 cm^−1^, corresponding to Amide I, is not observed, and the peak of 1571 cm^−1^, Amide II, coincides with the G^–^ peak from the carbon nanotubes. Such an overlap complicates a detailed assessment of the structural changes of components in experimental samples. Also, the frequency coincidence manifested itself at about 1350 cm^−1^—the torsional vibration of CH_3_ and CH_2_, which overlaps the D peak of the SWCNTs.

Secondly, no significant changes in the structure of the nanotubes were found. Unlike collagen, the “before and after” spectral dependences of the exposure to blood plasma reproduced all the characteristic peaks. There is a peak of ~ 170 cm^−1^—the RBM mode characteristic of single-walled (and low-walled) nanotubes. Also, the peak at about 1350 cm^−1^—D peak (disorder-induced) from SWCNTs. The G peak is represented by bands of 1570 cm^−1^—G^–^ peak (caused by vibrations perpendicular to the tube axis) and 1588 cm^−1^ G^+^ peak (caused by C = C vibrations along the axis of the tube), which corresponds to the manifestation of semiconductor properties by nanotubes. A comparison of the I_D_/I_G_ intensity, in this case, shows a low degree of defect in the SWCNTs. The second part of the spectrum is characterized by the presence of a band of 2688 cm^−1^—overtone, 2D, not associated with defectiveness. It is determined by the scattering of a photon by two phonons of the lattice with equal in the magnitude, but oppositely directed momenta, and, therefore, is observed even in defect-free samples. The peak 3195 cm^−1^—overtone, D + G, caused by defectiveness. 

Thirdly, we can confirm the previously expressed assumption on the decrease in the collagen component in the coating and the increase in the concentration of carbon nanotubes. This is evidenced by a change in the ratio of the intensities of the D and G peaks (red and black lines in Figure 4).

### 2.4. Wetting Angle

**Figure 5:**
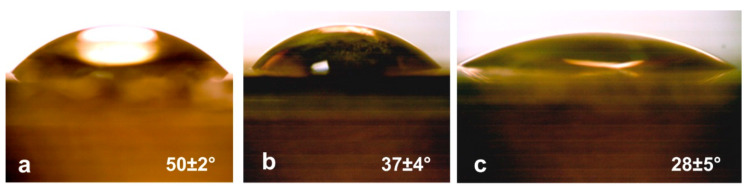
Appearance of (**a**) water droplets on the surface of PET tape, (**b**) PET tape coated with collagen, and (**c**) PET tap coated with SWCNT/collagen.

Using the SWCNT/collagen frame coating, the hydrophilicity of the PET tape was significantly improved. This is evidenced by studies of the wetting angle of the PET tape, the PET tape coated with collagen, and the PET tape coated with SWCNT/collagen. We considered the results of five repeated measurements (at five different points) for five samples of each type. The average values of the wetting angle are shown below (Figure 5). The figure shows that the PET tape coated with SWCNT/collagen had the smallest angle, which may be due to the presence of SWCNT functionalized in the carboxyl group.

For the PET tape coated with SWCNT/collagen, the largest scatter of values (error) was demonstrated. This is most likely due to the clear-cut surface topography, which depends on the topology of the SWCNT frame. Also, the calculation error for all samples was associated with the inhomogeneous surface of the PET tape due to the cellular plexus of the PET fibers. Thus, the surfaces of all the samples could be considered hydrophilic because the values of the wetting angles were less than 90°, but the coating SWCNT/collagen allowed to achieve a minimum wetting angle of 28 ± 5°.

### 2.5. Hemolysis

**Figure 6:**
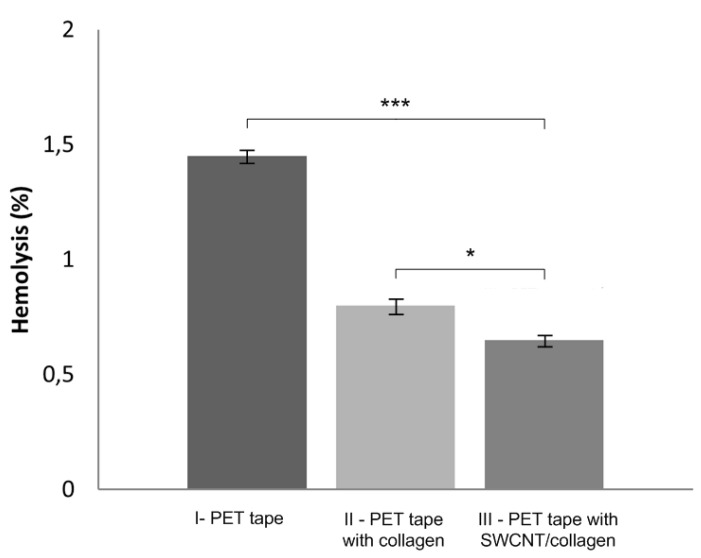
Hemolysis level chart for PET tape, PET tape coated with collagen and SWCNT/collagen. Statistical difference between percentage hemolysis was evaluated using the two-way ANOVA plus Tukey post-comparison test, where * denotes *p* ≤ 0.05 and *** denotes *p* ≤ 0.001.

In a hemocompatibility study of the biodegradable coating of SWCNT/collagen for a PET tape, the level of hemolysis was determined. The surface ability of the ligament implant to cause hemolysis was determined by the described method. Statistical significance was tested with two-way ANOVA. Hemolysis analysis for five control and five experimental samples showed that the PET tape provides 1.45 ± 0.052% hemolysis. Collagen and SWCNT/collagen-coated PET tape samples were non-hemolytic; hemolysis levels were reduced to 0.8 ± 0.063 and 0.65 ± 0.049%, respectively (Figure 6). The obtained hemolysis values, taking into account the calculation error, were lower than the criteria established by ASTM F756-08 for medical materials (ASTM F756-08 Standard practice for assessment of hemolytic properties of materials). Coated PET tape has a hydrophilic surface, which practically does not interact with blood components and prevents damage to red blood cells. Therefore, the proposed coating improves the hemocompatibility of PET. 

### 2.6. MicroCT at the Implantation Site

**Figure 7:**
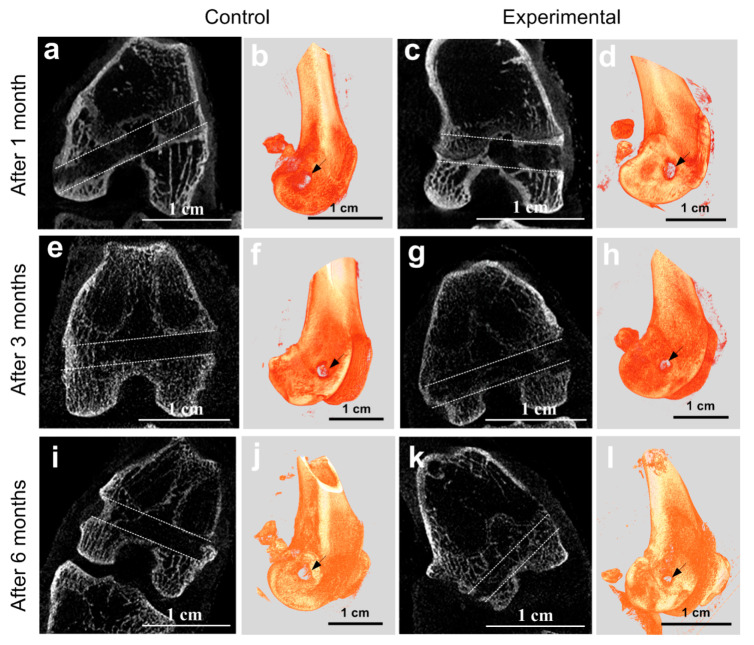
MicroCT projections (**a**,**c**,**e**,**g**,**i**,**k**) and three-dimensional images (**b**,**d**,**f**,**h**,**j**,**l**) of the transverse channel in the femur metaepiphysis for the control left and (**b**,**d**,**f**) experimental right knee joints through (**a**,**b**) 1, (**c**,**d**) 3, and (**e**,**f**) 6 months after implantation. All studies were conducted ex vivo.

To orientate in the internal structure of the bone channel with the implanted experimental and control ligament implants, ex vivo MicroCT imaging was made. In total, three control and three experimental joints of animals were studied for each of three periods after implantation (1, 3, and 6 months). The most informative images are shown in Figure 7. The ligament implants were practically not visible on the tomograms. Therefore, the process of osteoinductance could be judged by the size of the bone channel and the morphology of the tissues surrounding the channel. 

We performed three-dimensional visualization of the bone channel of each joint (Figure 7b,d,f,h,j,l). The arrow marks the channel entrance. Using these images, the dynamics of the change in the size of the hole at the beginning of the canal can be observed. The part of the canal that lies in the projection plane is clearly shown on tomographic projections (Figure 7a,c,e,g,i,k). For the visual representation, the contour of the channel is marked with a dotted line. The results showed that no significant changes in the canal region were observed in the experiment and control at one month after implantation. The channel was well visualized throughout and had clear boundaries. The tomograms (Figure 7a,c) show that the process of osteogenesis continues actively in all laboratory animals. Epiphyseal growth plates are clearly visible in the photographs. In the region of the pineal gland, the thin trabeculae of the cancellous bone are clearly distinguishable; they form a loose network around the implantation channel. 

Three months after implantation of the ligament in the femur, the metaepiphyseal growth plate was poorly visible, and the epiphyses were completely formed by the cancellous bone (the epiphyseal bone is completely formed) (Figure 7e,g). The tomogram shows that the plates of the spongy bone lie more densely than in the previous period. The boundaries of the channels in places are not clearly traced, but the lumen of the channel is viewed satisfactorily. In some places, trabeculae of the trabecular bone begin to sprout. In the bone channel with the implanted PET tape coated with SWCNT/collagen, this process was more intensive than that in the controls. The average diameter of the experimental bone channel was less than the control. Since implantation, the diameter has decreased from 2.5 ± 0.08 mm to 2.3 ± 0.06 mm. 

A similar pattern was observed six months after implantation on the three-dimensional image (Figure 7j,l) and tomographic projections (Figure 7i,k). In the femur, bone formation processes were completely completed. The boundaries of the channels were not visible in some places, and in some parts of the channel, the clearance was significantly reduced (Figure 7l). Trabeculae of the trabecular bone appeared in it. The diameter of the channels in the experimental and control samples was not uniform in length. In this case, the germination into the channel of the trabeculae in the case of an experimental sample was observed more often than in the controls. This has been observed on microCT projections (Figure 7a,c,e,g,i,k) and three-dimensional images (Figure 7b,d,f,h,j,l) of the transverse channel in the femur metaepiphysis. For controls, the bone channel diameter was 2.2 ± 0.1 mm. The diameter of the PET tape channel coated with SWCNT/collagen basically decreased to 1.7 ± 0.07 mm. This may indicate a more intensive process of channel overgrowing due to better osteoinductance of the SWCNT/collagen coating.

### 2.7. Histological Studies of Implantation Site

**Figure 8:**
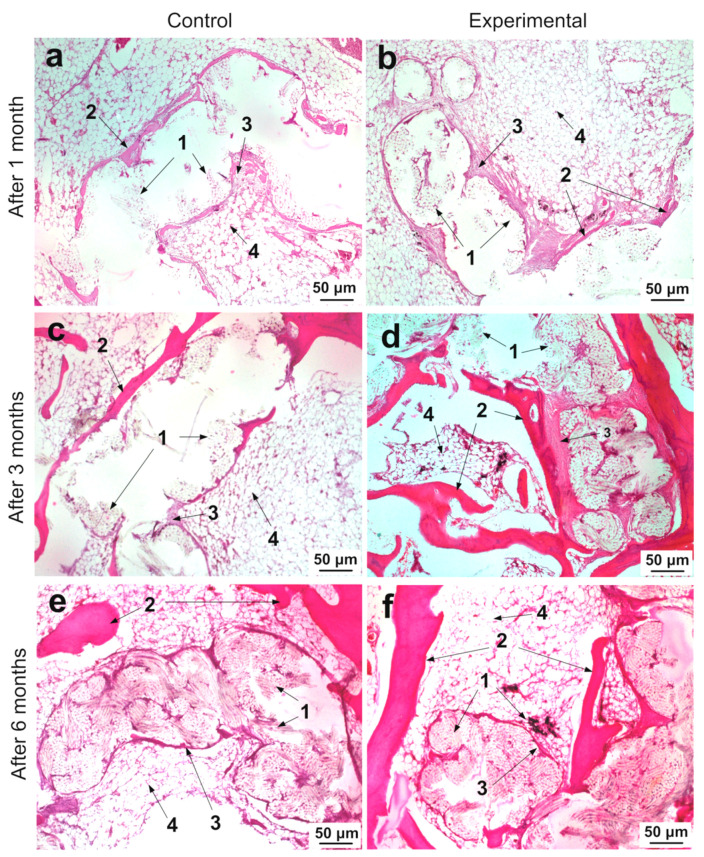
Images of histological sections in the implantation area after 1 (**a**,**b**), 3 (**c**,**d**), and 6 (**e**,**f**) months of the control (**a**,**c**,**e**) and a PET tape coated with SWCNT/collagen (**b**,**d**,**f**), where **1**—a PET tape fiber; **2**—bone trabeculae; **3**—connective tissue; **4**—reticular tissue.

**Figure 9:**
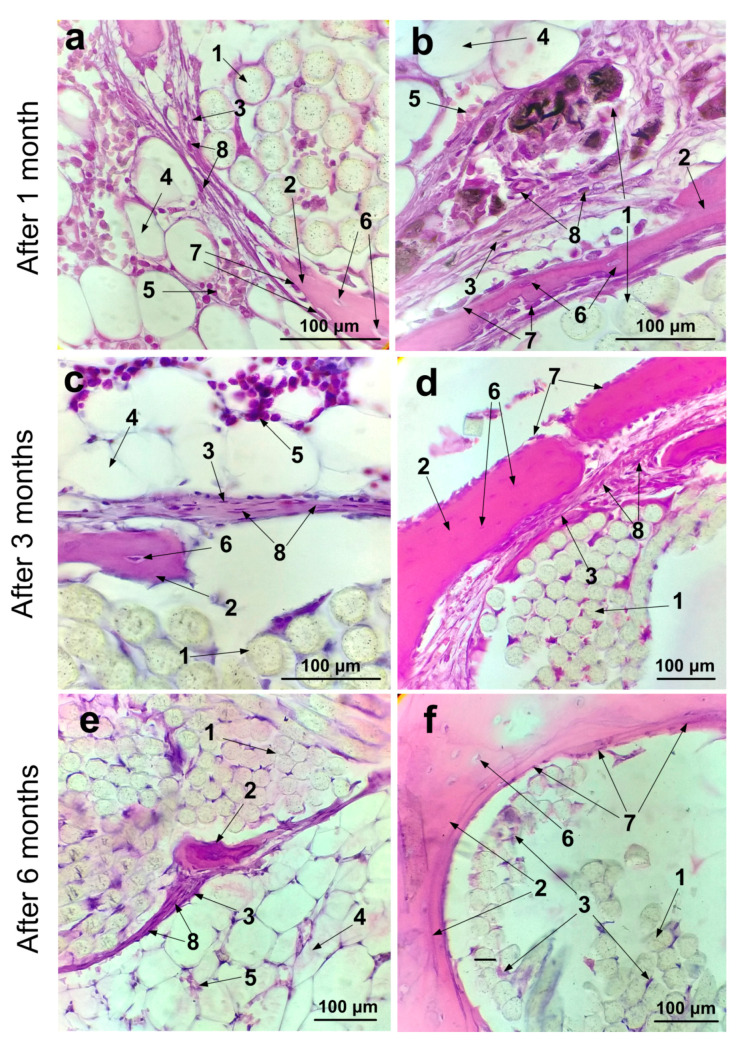
Enlarged images of histological sections with visualization of cells in the implantation area in 1 (**a**,**b**), 3 (**c**,**d**), and 6 (**e**,**f**) months of control (**a**,**c**,**e**) and a PET tape coated with SWCNT collagen (**b**,**d**,**f**), where **1**—a PET tape fiber; **2**—bone trabeculae; **3**—connective tissue; **4**—reticular tissue; **5**—blood cells at different stages of hematopoiesis; **6**—osteocytes; **7**—osteoblasts, 8—fibroblasts.

In contrast to MicroCT, histological examination allows a more detailed microscopic assessment of the state and structure of the epiphyseal bone, as well as the tissue and cellular response to implantation. Histological studies were mainly performed at a distance of 5–6 mm from the beginning of the channel along its direction (Figure 7b,d,f,h,j,l). It was necessary to match histological slices in a specific region of the channel for the control and experimental joints. Analysis of the implantation site of the three control and three experimental samples of ligament implants for each of the three periods after implantation showed the following histological pattern. At low magnification, it can be seen that thin trabeculae of the bone tissue formed around the perimeter of the bone channel (Figure 8a,b). It should be noted that osteocytes lying in the lacunae of trabeculae are spindle-shaped flattened cells. They are oriented along the axis of the bone channel (Figure 9a,b). The space between them is filled with reticular tissue, in which the elements inherent in the hematopoiesis process are identified. In this bone section, an active process of bone tissue formation was observed. Active osteoblasts were located in large numbers on the surface of bone trabeculae (Figure 9a,b). As can be seen in Figure 9b, these cells are in the active phase of excreting intercellular substance products. They are quite large, and they also have a rounded core with a predominance of euchromatin. A month after implantation, no traces of an inflammatory reaction were found in the control bone channel and in that with PET tape coated with SWCNT/collagen. During the healing process, the PET tape was located on the periphery of the channel. In the central area of the channel, fibrous connective tissue began to actively form, which filled the free space. It was well detected by the histochemical reaction. This pattern is characteristic of the control and experimental bone channels (Figure 8a,b). Mostly, connective tissue was represented by collagen fibers, and in some areas, cellular elements predominated. The main cellular form of the connective tissue was fibroblasts, which secrete components into the intercellular substance. They are located quite chaotically and are elongated cells, the boundaries of which are not visible on the preparations due to the peculiarities of staining the ectoplasm zone. Fibroblast nuclei are active and elongated (Figure 9a,b).

In the experimental channel, collagen fibers appeared more mature and formed thicker bundles than in the control sample (Figure 9a,b). Germination of collagen fibers between the PET tape fibers coated with SWCNT/collagen was also observed (Figure 9b). Images show small fibers with dark SWCNT/collagen coating.

Three months after implantation in the control and experimental joints, the surface of the trabeculae looked undisturbed and even (Figure 8c,d). A large number of active osteoblasts were located on the surface of the trabeculae (Figure 9c,d). Osteoclasts were practically not observed in the study periods. This indicates low destruction processes in the bone tissue. Slices on the surface and in the deep parts of the pineal glands demonstrated the development of connective tissue at the implantation site in the control and experimental samples. 

At the periphery of the epiphyse with an implanted SWCNT/collagen-coated PET tape, significant growth of dense connective tissue was shown (Figure 8d). More mature collagen fibers prevailed here than in the control sample (Figure 9d). In the central part of the epiphyse with a PET tape coated with SWCNT/collagen, the formation of bone trabeculae was more pronounced than in the control bone channel. At the same time, a certain amount of connective tissue (collagen fibers and fibrocytes) was observed between bone trabeculae and a PET tape coated with SWCNT/collagen (Figure 9d). The fixing process for the PET tape coated with SWCNT/collagen was as follows. Around the PET tape coating, a relatively thin layer of dense connective tissue was observed, which was surrounded by a continuous bone trabecula along the contour. This process was more pronounced in the experimental channel than in the control channel. In some areas, bundles with SWCNT/collagen-coated PET tape were outside the bone trabeculae. This probably occurs when bone tissue sprouts in the PET tape. In the experimental bone channel in the intercellular substance of the connective tissue, individual cells were observed, probably of the phagocytic series. In the control channel, a collagen-coated PET tape was fixed due to the active growth of the peripheral implanted connective tissue ligament, as well as the growth of the connective tissue between the bundles of fibers inside the bundle and between individual fibers. In the central region of the PET tape between and around the fibers, dense connective tissue was practically not observed or was present in a small amount (Figure 8c). In the free space of the channel, reticular tissue actively grew, and the border of the implantation channel was well defined (circled by a thin layer of bone tissue) (Figure 8c).

Six months after implantation of collagen and SWCNT/collagen-coated PET tape, a bone that was mature in all respects was observed. Trabeculae of cancellous bone in the central part of the femur epiphyse were present in smaller numbers but had become much thicker (Figure 8e,f). Mostly passive osteoblasts were found (Figure 9f). They differ from active osteoblasts by their smaller size and more flattened nucleus. Active osteoblasts were practically absent or were found in insignificant amounts (Figure 9f). Passive osteoblasts were found mainly on the surface of bone trabeculae from the side of PET fibers. Osteocytes were located in lacunae that were elongated along the growth direction of the trabeculae (Figure 9d,f). The layer of the compact substance by the nature of the structural components looked more mature. Bone plates, which form concentric structures—osteons, and insert plates located between osteons, were well pronounced. The space between the trabeculae was filled with reticular tissue with hemopoiesis islets. Bone tissue trabeculae were more developed in the experimental channel than in the control channel (Figure 8f). They surrounded the PET tape and were present in the cavity of the bone channel. A small amount of loose connective tissue was observed with a predominance of the cellular component (Figure 8f). In the control channel, bone trabeculae next to collagen-coated PET tape were less common. The bone channel was visually assessed as wider than the experimental one (Figure 8e). 

## 3. Discussion

Artificial ligaments based on PET fibers have shown a satisfactory clinical effect in the short, medium- and long-term follow-up period [17,62]. However, like many polymer or metal implants, PET ligaments can cause serious side effects, such as a long period of integration with soft tissues and bones, weakened attachment in the bone channel, and synovitis [63,64]. This is especially important in the first time after implantation (about six months). The lack of biological integration of PET is associated with its hydrophobic and inert properties [20]. The success of ligament reconstruction depends on the healing efficiency of the bone channel and fixation of the artificial ligament in it in the short term (the first few months after implantation). To ensure a biocompatible, cytophilic, osteoinductive effect, coatings for PET are used [26,27]. The addition of carbon nanotubes can improve the coating properties, as both increasing its mechanical rigidity, i.e., tensile strength, and forming a nanostructured surface that facilitates the cell adhesion [65]. We modified the artificial ligament by forming a SWCNT/collagen frame coating on the surface of the PET fibers to improve the biocompatibility and hemocompatibility of the implant in the first six months after the operation. A new coating method using spin coater and irradiation by infrared (IR) source allowed us to form a uniform and homogeneous SWCNT/collagen coating. Under the influence of IR radiation, it is noticeable that SWCNT and their bundles form a frame structure. It is known that, after synthesis, carbon nanotubes have a large number of defects, mainly vacancy defects [51]. Nanotubes also have high thermal conductivity; however, in the defect region due to the violation of the crystal lattice, thermal conductivity decreases, and hot spots form [53]. Such spots are the most probable regions of nanotube binding to each others frame structures [54]. In this case, collagen acts as a surfactant, preventing the adhesion of SWCNT, which inevitably arises under the influence of Van der Waals forces.

As shown in the study, the ability of a coating to biodegrade under the influence of body fluids (blood plasma) contributes to structural changes in the coating—an increase in the number of pores and their size, which is necessary for the cells to grow into the coating structure to ensure better adhesion and integration of the implant with surrounding tissues. The pores with such dimensions are necessary for the implementation of neovascularization and neoinnervation processes. This was ensured by the technological mode of coating formation using spin coater with simultaneous irradiation by the IR source.

The ligament implant must actively interact with the biological fluids and cellular elements after implantation; this is of particular importance in the early stages of osseointegration [65]. Therefore, another important aspect of the coating is its stability in liquid media. It is well known that when immersed in a liquid, collagen swells, absorbs, and retains a large amount of water [66,67]. Hydrophilicity is a decisive property for tissue regeneration since the degree of swelling of the material in a liquid medium in combination with the stability of the material will provide an interaction between the cells and the coating. Since the material SWCNT/collagen is composite, representing a collagen matrix reinforced with a carbon nanotube frame, the material has long-term stability with a high degree of swelling. This will enhance interaction between tissue cells and the implant over a long period of time (at least 3–6 months). In addition, after this period, the biomaterial can biodegrade and be reabsorbed by the body [65]. In the study of the contact angle, it was shown that with the help of a SWCNT frame, it was possible to significantly change the hydrophilicity of the PET tape upwards. This may be due to the presence of carboxyl groups on the surface of the SWCNT, reinforcing collagen. The surfaces of the implants with insufficient hydrophilicity could adversely affect the erythrocyte membranes, which could lead to the process of pathological hemolysis [68]. PET is known to have poor hemocompatibility, which can be improved by modifying the surface of the PET with bioactive coatings [69]. We have shown a reduced hemolysis level upon contact with the blood of a PET tape implant coated with SWCNT/collagen compared to the original PET tape and PET tape coated with collagen. The evaluation of hemolysis showed that the smallest hemolysis was observed for coating SWCNT/collagen (hemolysis 0.65 ± 0.049%), which was 2.2 times less than that of a non-coated PET implant (hemolysis 1.45 ± 0.052%). At the same time, collagen coating without SWCNTs made it possible to reduce hemolysis by 1.8 times (hemolysis 0.8 ± 0.063%).

The healing of the bone channel, in which the ligament implant is fixed, largely depends on the efficiency of the formation of a new bone and dense connective tissue around the implant. Therefore, it is important to enhance biological healing at the implant/bone border using growth factors, cell therapy, and various other methods of drug delivery and stimulation of regeneration. According to animal studies, osteoinductive growth factors, including BMP, transforming growth factor and fibroblast growth factor, have a positive effect on the restoration and healing of connective and bone tissues [70,71]. However, there are some drawbacks, such as the short service life, difficulties in storage and processing, complex synthesis, inefficiency of the recognition of target cells, and the high cost of their use [72,73]. Therefore, all this greatly complicates the operation. The ability of nanotubes to act as an osteoinducer has previously been demonstrated [74]. In addition, the interaction of nanotubes with osteoblasts was described, and good biocompatibility and bone formation were observed [75,76,77,78]. To use the potential of the nanotube frame for tissue engineering, it is extremely important to select a suitable matrix. Composite materials based on nanotubes and synthetic polymers (polyvinyl alcohol, polymethyl methacrylate, polylactic acid, etc.) were widely studied and showed good results [79,80]; however, the use of natural polymers in combination with nanotubes proved to be more suitable in terms of biocompatibility and the ease of functionalization of nanotubes with biomolecules [51].

Collagen, as one of the most common connective tissue proteins, plays a critical role in the structural support of tissues, cell adhesion and migration, the formation of integral tissues, and their remodeling [81]. When mixing collagen and CNTs, an improvement in the mechanical properties of the collagen matrix is observed. Bionanocomposite based on nanotubes and collagen is osteogenic, bioresorbable, and has the desired mechanical rigidity while maintaining a three-dimensional nanostructured surface. The stability and hydrophilicity of such a material can also be controlled by changing the concentration of nanotubes in the composite [65]. In our work, we used a SWCNT concentration of 0.01 wt.%, which, on the one hand, worked well for the frame formation, and on the other hand, did not cause cytotoxicity [51,82,83]. Comprehensive in vivo studies have provided a fairly complete picture of the implantation of PET tape coated with SWCNT/collagen. Differences in the response of the bone channel tissue to implantation of collagen-coated PET tape and SWCNT/collagen were revealed. We performed ex vivo imaging of samples by microCT to obtain the dynamics of the changes in the geometric shape of the bone channel along its entire length. According to the results of the histological studies one month after implantation, significant differences in the size of the bone channel and in the structure of the surrounding tissue between the control and experimental channels were not found. However, six months after implantation of a PET tape coated with SWCNT/collagen, a decrease in channel diameter from 2.5 ± 0.08 to 1.7 ± 0.07 mm was detected. Large bone trabeculae were visualized in some parts of the channel cavity. At the same time, the diameter of the bone channel with control samples decreased from 2.5 ± 0.08 to 2.2 ± 0.1 mm in 6 months after implantation. This observation may indicate osteoinductance of the SWCNT/collagen coating. Using microCT images, the characteristic regions for obtaining histological sections were determined to study the pattern of tissue repair. Using histochemical methods, it was possible to evaluate the composition and degree of maturity of the connective tissues, the structure of the bone tissue and the interaction of the PET tape with the surrounding tissues. Histological and histochemical analysis showed that in the case of the application of SWCNT/collagen coating to the surface of a PET tape, more active growth of connective tissue with mature collagen fibers was observed in the implantation area. One month after implantation, SWCNT/collagen coating stimulated the proliferation of fibroblasts, as we have previously obtained [51,54,84,85]. This is very important because imperfect cellular homeostasis is considered to be the reason for the impossibility of independent restoration of joint ligaments (immature fibroblasts that cannot form a connective tissue scar without the preliminary formation of the fibrin scaffold are produced in the area of ligament rupture). 

Towards the end of the experiment, connective tissue fibers mature, fibroblasts become fibrocytes, and the thickness of the collagen layer around the SWCNT/collagen-coated PET tape decreases. After three and six months of observation, the stimulating effect of SWCNT/collagen on the formation of bone trabeculae around the PET tape and, in general, in the cavity of the experimental bone channel became apparent. It is likely that after the ribbon-like ligament is located around the perimeter of the channel in the bone, the center of the channel is filled with a liquid medium, and then this cavity is overgrown with reticular tissue, which is then replaced by dense connective tissue. Connective tissue, in turn, is replaced by bone tissue. This is very important because the native connection of ligaments with bone is a highly specialized and organized tissue that transfers mechanical stress from soft tissues to bone [11]. This is consistent with the results of the accelerated early differentiation of osteoblasts on three-dimensional collagen scaffolds with multi-walled carbon nanotubes (MWCNT) compared to scaffolds from pure collagen [76]. Hirata et al. demonstrated the stimulation of new bone formation in the pores of collagen and MWCNT scaffolds as early as 28 days after implantation into the femur. One of the reasons for the active adhesion of osteoblasts to materials based on carbon nanotubes is the developed disordered nanostructure of the surface [61,77]. In addition, carbon nanotubes inhibit osteoclastic differentiation in vitro and osteoclastic bone resorption in vivo [78]. Thus, the tight adhesion of osteoblasts and their early differentiation into collagen and carbon nanotube scaffolds maintain the scaffold shape and enhance bone formation in accordance with the pore morphology of scaffolds.

## 4. Materials and Methods 

### 4.1. Modeling the Interaction of the Complex SWCNT/Collagen

**Figure 10:**
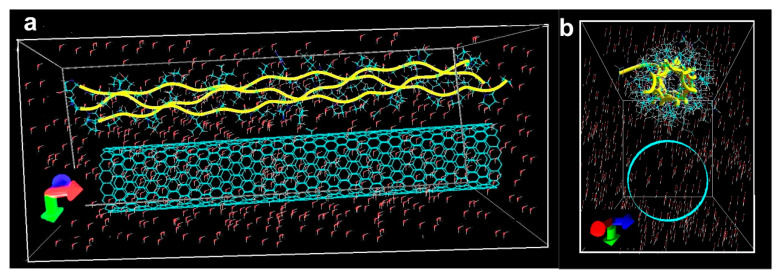
Atomistic model of the SWCNT (16,0)/collagen II (periodic water box) complex: (**a**) View of the model along the axis of the nanotube; (**b**) Cross-sectional view perpendicular to the axis of the nanotube (a triple-helix of collagen is highlighted in yellow). The arrows represent a tree-dimensional coordinate system (X – red arrow, Y – blue arrow, Z – green arrow).

The types of nanotubes that are most often synthesized by the electric arc method were considered as the SWCNT models. In particular, a thin nanotube (6,5) with a diameter of 0.75 nm, and a nanotube (16,0) with a diameter of 1.25 nm were considered [86]. They relate to the semiconductors, which make up 2/3 of the total number of synthesized SWCNTs. The tubes’ length was ~10 nm, respectively, of the length of type II collagen. The type II collagen structure was taken from the Protein Data Bank. An atomistic model of the initial configuration of the SWCNT complex (16,0) with collagen II is shown in Figure 10. The SWCNT/collagen complex is located in the aquatic environment. The periodic box has dimensions 10 × 5 × 6 nm, which contains 500 water molecules. Figure 10a shows the initial position of a triple-helix of collagen II (yellow curved lines) with respect to SWCNT, and triple-helix is located along the axis of the nanotube (X-axis). Figure 10b shows another view of the original model, where the cavity in the transverse section, formed by a triple-helix of collagen (perpendicular to the X-axis), is clearly visible.

The search for the configuration of the energetically favorable (equilibrium) mutual arrangement of the SWCNT and collagen was carried out in two stages.

(1) At the first stage, the molecular dynamics method using the AMBER force field [87], implemented in the HyperChem program (HyperChem Release 8.0.10, Hypercube, San Mateo, CA, USA), was used. The simulation time step was 1 fs; a thermostat was used to maintain the required temperature. The simulation time was 1–2 ns. At this stage, equilibrium SWCNT/collagen models are revealed at a given temperature as a result of the molecular dynamics modeling.

(2) At the second stage, the equilibrium models obtained at the first stage were applied. In this case, the well-tested quantum SCC DFTB method [88], successfully implemented in the DFTB + program, was used. The obtained equilibrium models SWCNT (6,5)/collagen II, and SWCNT (16,0)/collagen II were subjected to further optimization at this stage by the SCC DFTB method. This ensures I) the refinement of the atomic structure of the SWCNT/collagen complex at a given temperature; II) opens up the possibility of studying the electronic structure. Also, at this stage, it is possible to study the interaction energy of the SWCNT with collagen as a function of temperature, that is, on the energy of the external irradiating electromagnetic field absorbed by this medium.

### 4.2. Fabrication of PET Ligament Implant with SWCNT/Collagen Coating

As a basis for the implant, we used PET tapes for ligament reconstruction (PGTO SEVER, Saint Petersburg, Russia) made of threads with an average diameter of 30 μm, twisted into fibers with an average diameter of 1 mm. The tapes were 4 mm wide and 250 mm long. They went through the cleaning process in a 75% ethyl alcohol solution (Himabsolut, Moscow, Russia) for 4 h, then were washed with distilled and deionized water and dried in a bactericidal storage chamber with UV—emitting lamp (KBU-1, SKTB-SPU, Russia) at room temperature for 24 h.

**Figure 11:**
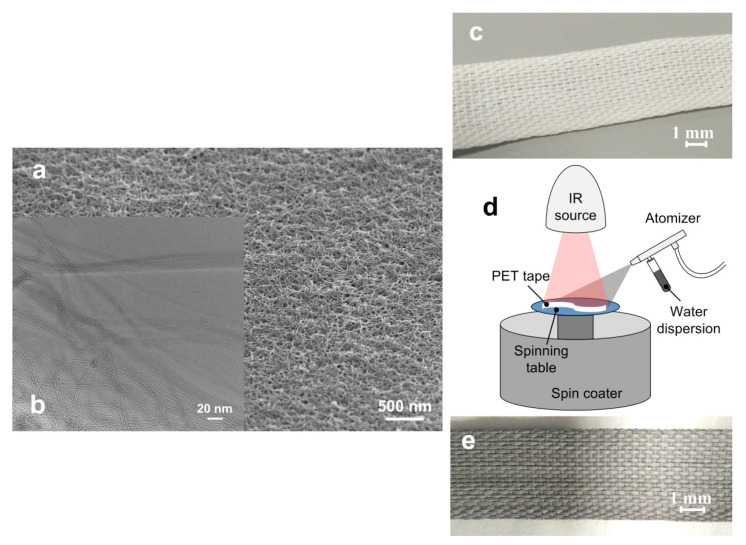
Images of (**a**) the SEM of the SWCNT overview map, (**b**) the TEM of the isolated SWCNT, and (**c**) the appearance of the PET tape fibers, (**d**) the installation diagram for forming the SWCNT/collagen coating on the PET tape and (**e**) the PET tape coated with SWCNT/collagen.

The following steps were taken to make the SWCNT/collagen dispersion. First, an aqueous suspension of high-purity type II collagen (MakMedi Ltd., Moscow Russia) extracted from the connective tissue of bulls was prepared with a concentration of ~2 wt.% with the addition of NaCl salt (0.5 wt.%). Registered tissue-engineering implants for ophthalmic surgery, cosmetology, dentistry, and other medical applications are made from such a suspension [89,90]. Next, an aqueous dispersion of SWCNT 2.5 wt.% was prepared. The SWCNT were synthesized by the electric arc method on a Ni/Y catalyst, purified in a dispersion of HNO_3_/H_2_SO_4_, with rinsing until neutral reaction after that (Scientific-Manufacturing Complex “Technological Centre», Russia). The average diameter of the nanotubes was about 1.4–1.8 nm, length—0.3–0.8 μm, and specific surface area was ~400 m^2^/g (Figure 11a). The purity of the SWCNT was 98%. SWCNTs were functionalized with carboxyl groups (–COOH).

To prepare the final dispersion, a collagen suspension and a SWCNT dispersion were mixed and treated alternately with an ultrasonic bath (Sapphire-TTS, Sapphire, Moscow, Russia) and a magnetic stirrer (C-MAG HS4, IKA, Staufen, Germany) for 5 h. During ultrasonic homogenization, the dispersion temperature was controlled in the range of 30–37 °C. The concentration of the final dispersion of SWCNT/collagen in the dispersion was 0.01 wt.% and 2 wt.%, respectively. 

To form a SWCNT/collagen coating on the PET tape, a spin coater (WS-400, Laurell Technologies, North Wales, PA, USA), and an infrared (IR) radiation source (YLR, IRE-Polus, Fryasino, Russia) were used. Recently, optical methods of forming solid biocompatible nanomaterials from liquids have proven themselves well [91,92]. Using a spin coater, a thin layer of dispersion was applied to the PET tape, which was mounted on a spin coater table. During the rotation of the sample, the dispersion was condensed using an IR source at a temperature of no more than 40 °C (Figure 11d). Then the next layer was applied, and the procedure was repeated. As a result, 5–7 layers were applied (Figure 11e). As a control sample, a ligament implant with a coating of collagen suspension was made (Figure 11c). All experimental and control samples were sterilized by ethylene oxide gas sterilization at 37 °C. Ethylene oxide is an alkylating agent that disrupts cellular metabolism and the reproductive processes of microorganisms. Ethylene oxide penetrates breathable packaging, making contact with all accessible surfaces of the product to deliver the required sterility assurance level. This method of sterilization was chosen because of the possibility of sterilization without exposure to high temperature and damaging the structure of the collagen by high-intensity radiation [93]. The quality control of the sterilization (the lack of growth of control microorganisms) was carried out using biological and chemical multiparameter indicators. As a biological indicator, we used that containing Bacillus atrophaeus spores attached to a strip placed in a container with a nutrient medium (Attest 1264, 3M, St. Paul, MN, USA). Ethylene oxide penetrates the container, killing spores, and thereby confirming compliance with the necessary conditions for sterilization. The chemical indicator is a strip that confirms the quality of sterilization by changing the color from red to green (Comply 1251, 3M, St. Paul, MN, USA). These indicators were selected due to the speed, ease, and accuracy of monitoring the quality of gas sterilization. 

The structure of the coatings was studied using an optical microscope (Eclipse L200, Nikon, Tokyo, Japan) and a scanning electron microscope (SEM) (Helios NanoLab 650, FEI Company, Hillsboro, OR, USA). The sample was fixed to an aluminum table using a conductive carbon adhesive tape to drain the charge from the sample. The studies were conducted under a high vacuum. The accelerating voltage of the electronic column was 5 kV. The current of the electron probe was 0.17 nA. Also, structural changes in the coating after contact with body fluids were investigated using Raman spectroscopy. Raman spectra were obtained on the spectrometer (LabRAM HR Evolution, HORIBA, France). The spectra were excited by a semiconductor laser with a wavelength of 633 nm, power up to 0.5 mW (depending on the signal-to-noise ratio), and a duration of 10 s. Spectra were measured at room temperature. The curves were averaged over 150 scans, recorded with a resolution of 1 cm^−1^, and subjected to the inverse Fourier transform.

### 4.3. Persistence and Weight Loss of SWCNT/Collagen Coating for the PET Tape in Blood Serum 

The resistance and weight loss of the coating were studied by the method used to test artificial ligaments from PET [94,95]. Thermal cycling of samples in physiological saline (Sigma-Aldrich, St. Louis, MO, USA) and blood serum was carried out. Since blood serum had a more aggressive effect on the coating, we will discuss it further. Before the start of the study, each of the 10 samples (a fragment of the PET tape coated with SWCNT/collagen) was weighed three times and placed in a vessel with blood serum. The vessel was placed in the environmental chamber (SH-641, Espec, Hudsonville, MI, USA). The serum was cooled and heated with a sample from 5 ± 1 to 55 ± 1 °C. Serum temperature was measured using an immersion temperature sensor. One cycle lasted about 3 min. Samples were thermocycled for 5000 cycles. This corresponded to six months of usage in the organism [96]. We deliberately chose such serious loads so that it could be said with confidence that the coating is not completely resorbed in the long run. The study of the changes in the structure of the samples was carried out using optical and electron microscopy. The relative weight loss of the samples after completion of thermal cycling was also evaluated. The relative mass loss was calculated as the percentage of the mass difference before and after thermal cycling to the mass before thermal cycling.

### 4.4. Wetting Angle of SWCNT/Collagen Coating

To determine the hydrophilic properties of the SWCNT/collagen coating, studies of the wetting angle were carried out. The wetting angle was calculated as the angle formed between the tangent to surface at the “liquid-coating” interface, and the contact point of the three-phase states liquid medium, material surface, and air. The measurements were carried out using a goniometer (LK-1, NPK, Moscow, Russia) according to the sessile drop method. A sample of the ligament implant was placed on a goniometer stage. Using a dispenser, a drop of deionized water with a volume of 1 μL was applied to the sample. The focus of the horizontal microscope was adjusted so that the droplet images and the boundaries between the droplet and the sample acquired clear and separate boundaries. Using a digital camera, which is part of the goniometer device, an image of a drop of liquid lying on the surface of the test sample, was obtained. Then, using the DropShape software (KRÜSS GmbH, Hamburg, Germany), the wetting angle was automatically determined by the tangent method. The measurements were carried out for the five experimental and five control samples at five different points on the surface.

### 4.5. Hemolysis Assay

Hemolysis studies are important for various types of implants, including orthopedic ones [68,97]. For the study of ligament implants with (experimental sample) and without (control sample) SWCNT/collagen coating, a solution of 4 mL of whole blood and 5 mL of a 0.9% aqueous solution of NaCl (normal saline) with a natural level of hemolysis was used. The hemolysis test was carried out under a strict protocol at I.M. Sechenov First Moscow State Medical University under the guidance of its Ethics Committee. Each of the 5 control and 5 experimental samples (1 × 0.4 cm^2^) was added to the solution with natural hemolysis for 30 min at 37 °C. Next, 0.1 mL of diluted blood was added to the samples and incubated for 2 h at 37 °C. As negative controls, a solution with a natural level of hemolysis was used (hemolysis—0%). As positive controls, a blood solution of 0.1 mL in 5 mL of deionized water was used after cooling with complete hemolysis (hemolysis—100%). After incubation in tubes, plasma and blood cells were separated by centrifugation at 1500 rpm for 10 min. The plasma from the tubes was added to quartz cuvettes to measure absorption spectra using a spectrophotometer (Genesys 10S UV-VIS, Thermo Fisher Scientific, Waltham, MA, USA) with a resolution of 0.2 nm relative to an empty cuvette. By comparing the optical density, the amount of free hemoglobin in the plasma was estimated. To quantify the hemolysis level, the values of the optical density of the plasma at a wavelength of 545 nm were obtained and the relative hemolysis δ (г) in % was calculated using the formula:

(1) $$\delta \left(г\right)=\frac{D_{\mathrm{sample}}\;-{\;D}_{\mathrm{control}}\left(0\;\%\right)}{D_{\mathrm{control}}\left(100\;\%\right)\;-{\;D}_{\mathrm{control}}\left(0\;\%\right)}\times 100\;\%$$

where Dsample—optical density of plasma at a wavelength of 540 nm after contact with the sample; Dcontrol (100%)—optical density of plasma with complete hemolysis (positive control); Dcontrol (0%)—optical density of plasma with natural hemolysis (negative control).

### 4.6. In vivo Experiment Procedure

Animal trials were carried out in accordance with institutional guidelines/protocols in agreement with national laws and policies for animal care. The guidelines were approved by M.Vladimirsky Moscow Regional Research Clinical Institute Moscow, Russia. The test involved nine laboratory rabbits, 1.5 years of age. The implantation of a ligament implant fragment was carried out in the stifles. 

A transverse tunnel (channel) was formed in the frontal plane of the metaepiphysis of the femur of the stifle. The diameter of the channel was 2.5 ± 0.08 mm. Next, the ligament implant was inserted into the bone channel. The implant was fixed due to its close contact with the walls of the bone channel. The left knee joint in the animal was experimental; a PET implant with SWCNT/collagen coating was implanted in it. The right stifle was a control; a PET coated with collagen was implanted in it. The experimental and control samples were implanted into the bone flush leveled using the same technique, taking into account the anatomical features of the asymmetry of the body. Next, soft tissues were sutured and processed according to standard methods. Since it was important to study the healing of the bone channel in the long term, laboratory animals were pulled out from the experiment 1, 3, and 6 months after implantation of three animals at a time. 

### 4.7. MicroCT Analysis of the Implantation Site

**Figure 12:**
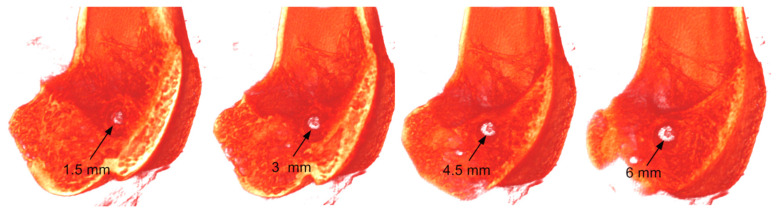
Slices of three-dimensional microCT images metaepiphysis femur with a transverse bore through 1.5, 3, 4.5, and 6 mm from the start of channel.

After the animals were pulled out of the experiment, ex vivo MicroCT analysis of the internal structure of the connection of the experimental and control implants with the bone channel was performed 1, 3, and 6 months after implantation for each animal. Eighteen fragments of the joint with implanted control and experimental samples were surgically removed and placed in flasks transparent for x-ray radiation on a slide of a SkyScan 1174 MicroCT (Bruker, Belgium). Scanning was carried out with the following parameters: voltage at the cathode of the x-ray tube—26 kV; current strength at the cathode of the x-ray tube—400 mA; current power at the cathode of the x-ray tube—9 W; camera resolution—1304 × 1024; rotation step—0.15 (about 4000 shadow projections). For reconstruction, data taken in the range of 180° were selected. Three-dimensional visualization of the obtained images was carried out in the CTvox software (Bruker, Belgium). The scan voxel size was ~30 μm. The obtained three-dimensional images allowed us to orient the sample for histological studies. The histological studies were done at approximately the same distance from the beginning of the channel along its direction for all samples. The characteristic area of the inner part of the channel was selected by scanning through a three-dimensional image of the metaepiphysis femur. This area was approximately 5–6 mm from the beginning of the channel (Figure 12).

### 4.8. Microstructural Analysis of the Tissue Morphology

For a microstructural analysis of tissue morphology, we used nine experimental and nine control samples of joints with ligament implants (six in one month after implantation, six in three months, and six in six months). Tissues were decalcified in an electrolyte acid solution (Sigma Aldrich) and washed for 24 h. Using a three-dimensional model of joint fragments with implants obtained using computer microtomography, samples were cut for histological analysis.

The manufacture of histological preparations from joints was carried out according to standard methods [69]. The histological sections were 5–7 µm thick. The sections of all tissues were stained with hematoxylin-eosin to obtain the overview preparations. To study the tissue structures in the area of the implanted ligament, histochemical staining of bone sections was carried out according to Van Gieson and Mallory [98]. Studies of the stained preparations were carried out using an optical microscope.

## 5. Conclusions

The study demonstrated a method for forming SWCNT/collagen coating for artificial ligaments made of the PET fibers. Such a coating is necessary so that the ligament fixes most tightly as a result of the healing of the bone channel in the short term after implantation. Electron microscopy was used to demonstrate the frame structure of the SWCNT in a collagen matrix, which is formed in layers by the phase change of an aqueous dispersion of SWCNT with collagen during spin coating and controlled irradiation with IR radiation. Equilibrium configurations of atomistic models of SWCNT/collagen type II complexes with nanotubes of different chirality (6.5) and (16.0) were obtained. The molecular dynamics method, in combination with the AMBER force field, revealed the nature of the change in the potential energy of the SWCNT/collagen complexes over 1–2 ns at various temperatures. Using the quantum-mechanical SCC DFTB method, it was first established that the highest binding energy between SWCNT and collagen was observed at temperatures of 303–315 K. In this case, the optimum temperature at which the maximum binding energy was reached was determined by the diameter of the SWCNT. For complexes with thin SWCNT of subnanometer diameter, the optimum temperature was less than for complexes with thin SWCNT of nanometer diameter. It was also found that the largest charge transfer was observed in the SWCNT/collagen complexes with larger diameter nanotubes. In the same complexes, the binding energy of SWCNT with collagen is also higher in comparison with complexes based on thin tubes. The formation of equilibrium SWCNT/collagen complexes occured already in the first 100–200 ps. In this case, the binding energy was determined primarily by the temperature, and simultaneously by the geometric parameters of the nanotube. The coating had a porous structure, the size of which ranged from 0.5 ± 0.01 to 6 ± 0.03 μm. The coating thickness was 20 ± 1 μm. SEM and Raman studies had shown a decrease in the coating mass and volume with initial collagen biodegradation after interaction with the blood plasma. This is proved by exceeding the G and D peaks intensity in the Raman spectrum after contact with blood serum and the decrease in the intensity of the main collagen band vibrations in the composition of the SWCNT/collagen frame coating. The size of the pores increased to 20 ± 0.5 μm. The modification of the PET tape with SWCNT/collagen coating made it possible to increase its hydrophilicity by 1.7 times in comparison with the original PET fibers and 1.3 times as compared with the collagen coating. Studies of the contact angle showed a greater variation in the error of the contact angle for the SWCNT/collagen coating due to its considerable relief on the PET fibers. This allowed a reduced hemolysis of the PET tape coated with SWCNT/collagen. The smallest hemolysis was observed for the coating of SWCNT/collagen (hemolysis of 0.65 ± 0.049%), which was 2.2 times less than that of a non-coated PET implant (hemolysis of 1.45 ± 0.052%). At the same time, collagen coating without SWCNT reduced hemolysis by 1.8 times (hemolysis 0.8 ± 0.063%). MicroCT has shown the effective formation of new bone and dense connective tissue around the implant. Six months after implantation of a PET tape coated with SWCNT/collagen, a decrease in channel diameter from 2.5 ± 0.08 to 1.7 ± 0.07 mm was detected. The diameter of the bone channel with control samples decreased from 2.5 ± 0.08 to 2.2 ± 0.1 mm in six months after implantation. MicroCT images were used to identify sites for histological sections, which demonstrated the degree of interaction of the PET tape with surrounding tissues. In the case of applying SWCNT/collagen coating to the PET tape surface, in the implantation area, more active growth of the connective tissue with mature collagen fibers was observed than in the case of coating only from collagen. After three and six months after implantation, the stimulating effect of SWCNT/collagen on the formation of bone trabeculae around and inside the PET tape and, in general, in the cavity of the experimental bone canal became apparent. Thus, it was found that the PET tape coated with SWCNT/collagen has osteoconductivity, as well as hydrophilicity and hemocompatibility.
